# Machine Learning-Based Classification of Gliomas and Tumor Grades with SHAP-Guided Feature Interpretation

**DOI:** 10.3390/genes17050511

**Published:** 2026-04-25

**Authors:** Ghaya Al-Rumaihi, Md. Shaheenur Islam Sumon, Ahmed Hassanein, Marwan Malluhi, Sakib Abrar Hossain, Tahmid Zaman Raad, Muhammad E. H. Chowdhury, Rozaimi Razali, Shona Pedersen

**Affiliations:** 1 Neurosurgery Department, Hamad Medical Corporation, Doha 3050, Qatar; galrumaihi@hamad.qa; 2 Department of Electrical Engineering, Qatar University, Doha 2713, Qatar; sumon3455.ms@gmail.com (M.S.I.S.); tahzam406@gmail.com (T.Z.R.); 3 College of Medicine, Qatar University, Doha 2713, Qatar; ah2309067@qu.edu.qa (A.H.); mm2308026@qu.edu.qa (M.M.); 4 Department of Biochemistry, University of Regina, Regina, SK S4S 0A2, Canada; mah690@uregina.ca; 5 Department of Biomedical Sciences, College of Health Sciences, Qatar University, Doha 2713, Qatar; rozaimi.r@qu.edu.qa

**Keywords:** glioma, glioblastoma, astrocytoma, oligodendroglioma, brain tumor, gene expression, genes, machine learning, artificial intelligence, SHAP analysis

## Abstract

**Background**: Gliomas are among the most common and heterogeneous primary brain tumors, exhibiting substantial molecular and transcriptomic diversity that complicates diagnosis, grading, and treatment planning. Advances in artificial intelligence (AI), particularly machine learning (ML), offer powerful opportunities to analyze high-dimensional gene expression data and support precision oncology. **Methods:** This study proposes an interpretable ML framework to classify brain tumor subtypes—glioblastoma, astrocytoma, and oligodendroglioma—and to predict tumor grades (2, 3, and 4) using microarray-based gene expression data. The analysis was conducted on the REMBRANDT dataset, comprising 464 labeled samples (221 glioblastoma, 148 astrocytoma, 67 oligodendroglioma, and 28 controls) and 314 tumor samples for grade classification. **Results:** The ML models achieved high performance for disease classification, with accuracies of 99.6% (AUC 99.89%) for glioblastoma, 98.3% (AUC 99.83%) for astrocytoma, and 98.95% (AUC 100%) for oligodendroglioma. Tumor grade predictions also performed strongly, achieving 83.7% accuracy (AUC 88.2%) for grade II vs. III, 91.3% (AUC 94.8%) for grade II vs. IV, and 84.2% (AUC 90.8%) for grade III vs. IV. SHAP analysis identified key genes contributing to the model predictions (e.g., *WIF1, STX6, RGS5*, and *ACTR2*), and KEGG enrichment identified the candidate pathways involved in vesicular transport, metabolism, and immune signaling. **Conclusion:** Overall, our findings demonstrate that interpretable ML models can accurately differentiate glioma subtypes and grades, and SHAP analysis can help identify the strongest predictors of our models. These findings provide additional insights into the heterogeneous genetic and molecular landscape of brain gliomas and are intended to complement, not replace, conventional histopathological diagnosis.

## 1. Introduction

Gliomas represent the most prevalent primary brain tumor within the central nervous system (CNS), arising from glial cells (i.e., astrocytes, oligodendrocytes, and ependymal cells), which provide crucial support and insulation for neurons [1]. Clinically, gliomas are divided into a range of subtypes, including oligodendrogliomas, astrocytomas, and glioblastomas, which differ in origin, molecular alterations, histopathology, and prognosis [2]. Oligodendrogliomas arise from oligodendrocytes and are characterized by isocitrate dehydrogenase (*IDH*) mutations and 1p/19q codeletion, which are associated with better treatment response and prognosis [3]. Astrocytomas originate from astrocytes and range from low-grade (grade I) to high-grade (grade IV) tumors. Grade I pilocytic astrocytomas are typically benign and occur in children, whereas grades II and III (diffuse and anaplastic astrocytoma) are infiltrative, malignant, and highly aggressive [4]. Glioblastomas (previously termed “Glioblastoma multiforme”) are defined as grade IV, *IDH*-wildtype astrocytomas [2,5], with the absence of an *IDH* mutation conferring a significantly poorer prognosis [6].

Glioblastoma remains the most aggressive and lethal glioma in adults, with limited improvement in survival despite advances in surgery, radiotherapy, and chemotherapy [7,8]. Its pronounced molecular and genetic heterogeneity contributes to variable responses to therapy and presents major challenges for precision medicine [9,10]. High-throughput sequencing technologies, such as single-cell RNA sequencing (scRNA-seq), have enabled detailed profiling of individual tumor cells, capturing transcriptional and genetic heterogeneity that is not fully detectable using conventional microarrays or quantitative PCR [11,12].

Artificial intelligence (AI) methods, including machine learning (ML) and deep learning (DL), offer powerful tools for analyzing these high-dimensional datasets, uncovering complex patterns, and identifying novel therapeutic targets [13,14,15,16,17,18,19,20,21]. Several studies have demonstrated the promise of ML/DL in glioma research [15,16,17,18,19,20,21]. Babaei et al. [15] used DL models to predict survival in glioblastoma patients and highlighted the importance of interpretability via Shapley Additive Explanations (SHAP), identifying age at diagnosis as a key predictor. Li et al. [22] applied autoencoders and XGBoost to classify glioblastoma versus osteosarcoma, revealing key gene signatures, such as *C2orf72*, with potential diagnostic and therapeutic value. Habibi et al. [16] reviewed DL approaches using radiomics for survival prediction in glioblastoma, noting both high variability and the need for standardized datasets. Suita et al. [17] leveraged ML and DL to model epigenetic regulation, identifying H3K27Ac as a critical predictor of transcript expression in glioblastoma stem cells. Thomas et al. [18] developed GBMPurity, a DL model estimating tumor purity from bulk RNA-seq with high accuracy, revealing subtype-specific differences. Hodeify et al. [19] combined metabolomics with ML to predict tumor status post-relapse, achieving 92% accuracy. Additionally, Drozdz et al. [20] and Cai et al. [21] demonstrated ML-based feature selection methods to classify astrocytoma grades and identify grade-specific gene signatures.

Building on these findings, our study applies machine learning models to the REMBRANDT gene expression dataset comprising 464 samples, including glioblastoma, astrocytoma, oligodendroglioma, and control (non-tumor) samples, with tumor-grade analyses focused on grades II–IV. Machine learning classifiers, including MLP, XGBoost, Logistic Regression, and Random Forest, were applied to perform binary classification between control and tumor types (glioblastoma, astrocytoma, oligodendroglioma) and across tumor grades (grades 2, 3, and 4). We also applied SHAP to identify key gene features contributing to model predictions, and gene set enrichment analysis (GSEA) was conducted to investigate the biological pathways associated with the identified genes. This ML framework is designed to serve as a complementary tool to standard histopathological diagnosis while providing additional insights into the complex and heterogeneous genetic nature of brain gliomas.

## 2. Methods and Materials

### 2.1. Study Population

The REMBRANDT (REpository for Molecular BRAin Neoplasia DaTa) dataset, a comprehensive collection of genomic and clinical data from brain cancer patients, supporting large-scale molecular and diagnostic research, was used in our study [23]. The dataset included genome-wide microarray-based gene expression profiles along with metadata such as gender (326 males, 177 females, and 168 unknown), disease types (e.g., 261 glioblastoma, 170 astrocytoma, 86 oligodendroglioma, 31 control (non-tumor)), and WHO tumor grades (2 grade I, 110 grade II, 93 grade III, 140 grade IV, with 326 missing).

### 2.2. Sample Collection and Processing

Tumor tissues were collected with ethical approval, and total RNA was extracted using TRIzol from each sample [23]. RNA quality was verified using the Agilent Bioanalyzer, after which high-quality RNA was analyzed using Affymetrix microarray chips. The resulting data were then quality checked, normalized, and preprocessed to obtain reliable gene expression values for further analysis.

### 2.3. Classification Strategy

Two types of analyses were performed using the REMBRANDT gene expression dataset. First, a disease classification task was conducted to distinguish brain tumor types—glioblastoma (*n* = 221), astrocytoma (*n* = 148), and oligodendroglioma (*n* = 67)—from control (non-tumor) samples (*n* = 28). After data preprocessing and cleaning, including the exclusion of samples with significant missing (NaN) values and standard scaling of the gene expression features, a total of 464 labeled samples were retained for analysis, and 5-fold cross-validation was applied to ensure robust model evaluation.

Second, tumor grade classification was performed to investigate gene expression differences across glioma progression stages. Grade I samples were excluded due to their limited representation (*n* = 2), and samples with missing or ambiguous grade annotations were removed. The final dataset included 314 tumor samples (99 grade II, 85 grade III, and 130 grade IV), and binary classification was conducted for the following grade pairs: grade II vs. grade III, grade II vs. grade IV, and grade III vs. grade IV.

### 2.4. t-Distributed Stochastic Neighbor Embedding (t-SNE)

To explore the inherent structure of the gene-expression data and assess class separability, t-SNE was applied to project the high-dimensional data into lower dimensions while preserving local relationships. Separate t-SNE plots were generated for disease classification (glioblastoma, astrocytoma, oligodendroglioma, and control) and tumor-grade classification (grades 2–4), using the top-ranked gene features. Corresponding t-SNE plots were provided in the Supplementary Materials Figures S1 and S2.

### 2.5. Dataset Preprocessing

The REMBRANDT gene-expression dataset was cleaned to remove missing values, and gene names were extracted from the microarray data. Features were normalized using StandardScaler [24] to ensure proportional contribution during model training. Five-fold cross-validation was performed using an 80% training and 20% testing split for both disease and tumor-grade classification tasks. Due to significant class imbalance, the training dataset was refined using the SMOTE-TomekLink technique [25]. SMOTE generated synthetic samples for the minority class, while TomekLinks removed overlapping instances near the decision boundary, effectively improving class balance.

### 2.6. Feature Selection

The gene expression dataset comprises 16,383 features, making effective feature selection a crucial step before applying machine learning algorithms. To identify the most relevant features, we employed XGBoost, Random Forest, and ExtraTrees-based feature selection methods, and based on each model’s performance, the best ones were selected for analysis.

### 2.7. Evaluation Metrics

Each ML model was assessed using multiple performance evaluation metrics, including the area under the receiver operating characteristic curve (AUC-ROC), precision, sensitivity, specificity, accuracy, and F1 score, to provide a comprehensive assessment of the performance of each model. The mathematical formulations of these metrics are presented in the supplementary “Evaluation Metrics” section.

### 2.8. Construction of ML Models

Ten machine-learning models were initially trained, encompassing a range of algorithmic families, including tree-based methods [26] (CatBoost, Random Forest, ExtraTrees, Gradient Boosting, XGBClassifier), ensemble techniques (LGBM) [27], linear models (Logistic Regression, ElasticNet) [28], and neural networks (MLPClassifier) [29]. Tree-based models performed particularly well on this gene expression dataset due to their ability to capture non-linear relationships and complex feature interactions.

In the first stage, we performed a set of binary classification tasks comparing the control group with glioblastoma, astrocytoma, and oligodendroglioma cases. In the second stage, we classified tumor grades 2, 3, and 4.

### 2.9. Pathway Enrichment Analysis

To investigate the biological significance of the top altered genes, pathway enrichment analysis was performed using Enrichr with the KEGG 2021 human pathway database [30]. This analysis identified disrupted signaling pathways and cellular processes associated with glioblastoma progression, treatment resistance, and immune evasion.

### 2.10. Experimental Setup

The investigation was conducted using the Scikit-learn package [31] with Python 3.10. All models were trained on a system equipped with an Intel Core i9 10th generation processor and an NVIDIA RTX 2080 GPU with 8 GB of memory. For pathway analysis, we used the Enrichr platform [30], and to identify associated diseases, we utilized DisGeNET [32] and Harmonizome 3.0 [33].

**Figure 1:**
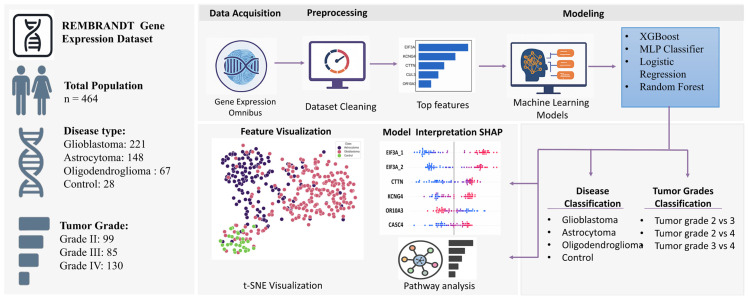
A workflow diagram of the gene expression-based machine learning pipeline for glioma subtype and tumor grade classification.

Figure 1 presents a detailed overview of the proposed analytical methodology utilizing gene expression profiling for the classification of glioma subtypes and tumor grades. The research employed the REMBRANDT gene expression dataset, which consists of 397 samples: glioblastoma (*n* = 221), astrocytoma (*n* = 148), oligodendroglioma (*n* = 67), and control (*n* = 28) tissues. Tumor grades II, III, and IV were also included. The workflow initiates with data collecting from the Gene Expression Omnibus (GEO), followed by preprocessing, which includes dataset cleaning and top feature selection.

Feature visualization was performed using t-SNE, enabling an intuitive exploration of class separability. Model training was conducted using a combination of machine learning classifiers, including MLP, XGBoost, Logistic Regression, and Random Forest, etc. These base learners were further integrated using a stacking-based meta-classifier. Model interpretability was enhanced using SHAP (Shapley Additive Explanations) to identify key gene features contributing to predictions. Importantly, binary classification was performed in both stages of analysis: (1) distinguishing between control and individual tumor types (glioblastoma, astrocytoma, and oligodendroglioma), and (2) comparing tumor grades in a pairwise fashion (grade 2 vs. grade 3, grade 2 vs. grade 4, and grade 3 vs. grade 4). Additionally, gene set enrichment analysis (GSEA) was performed to contextualize the biological relevance of the selected features within known functional pathways. Moreover, hierarchical clustering heatmaps using top-ranked genes (Figures S3–S5) were generated to illustrate gene expression patterns across the four tissue types and the three tumor grades, revealing clusters of co-expressed genes and similarities among the samples.

## 3. Results

### 3.1. Feature Selection Results

**Figure 2:**
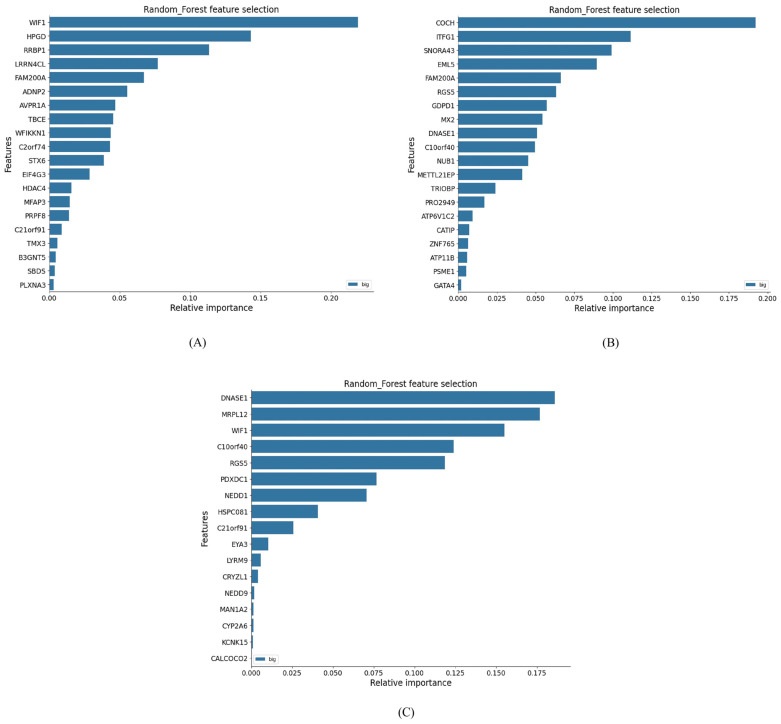
The top features using Random Forest feature selection. (**A**) Control vs. glioblastoma, (**B**) control vs. astrocytoma, and (**C**) control vs. oligodendroglioma. The bar plots display the relative importance of selected genes, highlighting their discriminative power in each case.

For disease classification (gliomas vs. control), Random Forest-based feature selection provided the best performance (Figure 2). The top-ranked features differed across tumor types: the highest classification performance for glioblastoma and astrocytoma was achieved using the top seven features, while six features sufficed for oligodendroglioma.

**Figure 3:**
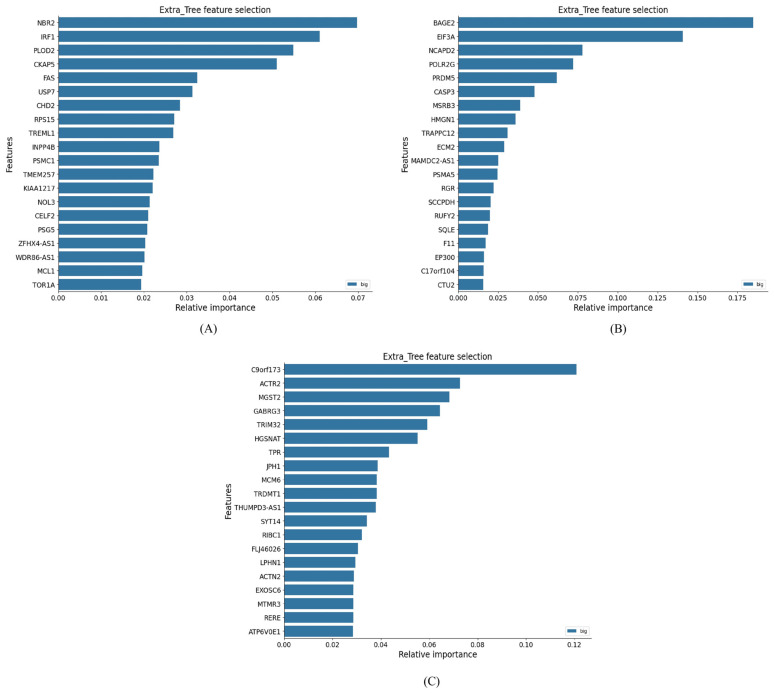
Top features for tumor grade classification using ExtraTrees. (**A**) Grade 2 vs. 3, (**B**) grade 2 vs. 4, and (**C**) grade 3 vs. 4. In each case, the features are ranked by their relative importance scores, reflecting their contribution to distinguishing between tumor grades.

For tumor grade classification, ExtraTrees-based feature selection performed best. Distinct gene sets emerged for each grade comparison, highlighting molecular differences across tumor progression that could serve as potential biomarkers (Figure 3). Specifically, the optimal feature sets included 20 genes for grade II vs. III, nine genes for grade II vs. IV, and 23 genes for grade III vs. IV.

### 3.2. ML Model Results

For disease classification (control vs. gliomas), multiple ML models were evaluated across three binary scenarios: control vs. glioblastoma, control vs. astrocytoma, and control vs. oligodendroglioma (Table 1). In the control vs. glioblastoma scenario, Random Forest (99.60%) and AUC (99.89%) achieved the highest accuracy, followed closely by XGBoost and Logistic Regression. For control vs. astrocytoma, Random Forest achieved 98.30% accuracy, while XGBoost recorded the highest AUC of 99.83%. In the control vs. oligodendroglioma comparison, SVM performed best, with 98.95% accuracy and 99.89% AUC.

For tumor grade classification (grade 2 vs. grade 3, grade 2 vs. grade 4, and grade 3 vs. grade 4), model performance is summarized in Table 2. Random Forest performed best for grade 2 vs. grade 3 (accuracy 83.70%, AUC 88.21%) and grade 3 vs. grade 4 (accuracy 84.19%, AUC 90.81%), while CatBoost slightly outperformed others in grade 2 vs. grade 4 (accuracy 91.27%, AUC 94.76%). Across all grade comparisons, Random Forest consistently showed robust performance in accuracy, F1-score, and AUC, while ExtraTrees and SVM were competitive, and AdaBoost and XGBoost generally trailed.

### 3.3. ROC Curves

**Figure 4:**
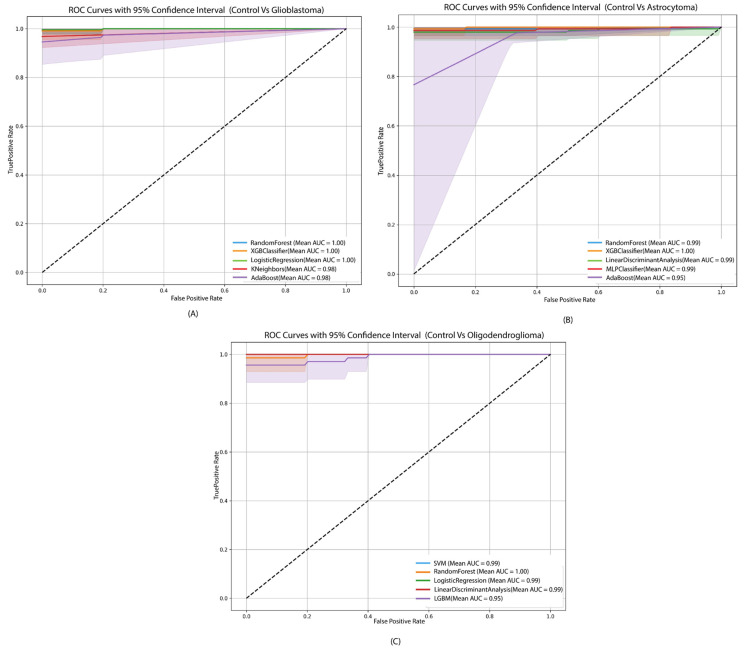
ROC curves with 95% confidence intervals for the top five performing machine learning models for the three disease classification scenarios. (**A**) control vs. glioblastoma, (**B**) control vs. astrocytoma, and (**C**) control vs. oligodendroglioma. The ROC curves shown correspond to the top five models identified based on their classification performance.

The ROC curves and AUC values were calculated for each ML model in disease classification (gliomas vs. control) and tumor grade comparisons (grades 2–4). For disease classification (Figure 4), Random Forest, XGBoost, and Logistic Regression consistently achieved near-perfect AUCs. In control vs. glioblastoma and control vs. oligodendroglioma, several models reached an AUC of 1.00, demonstrating a highly accurate separation between tumor and control samples.

**Figure 5:**
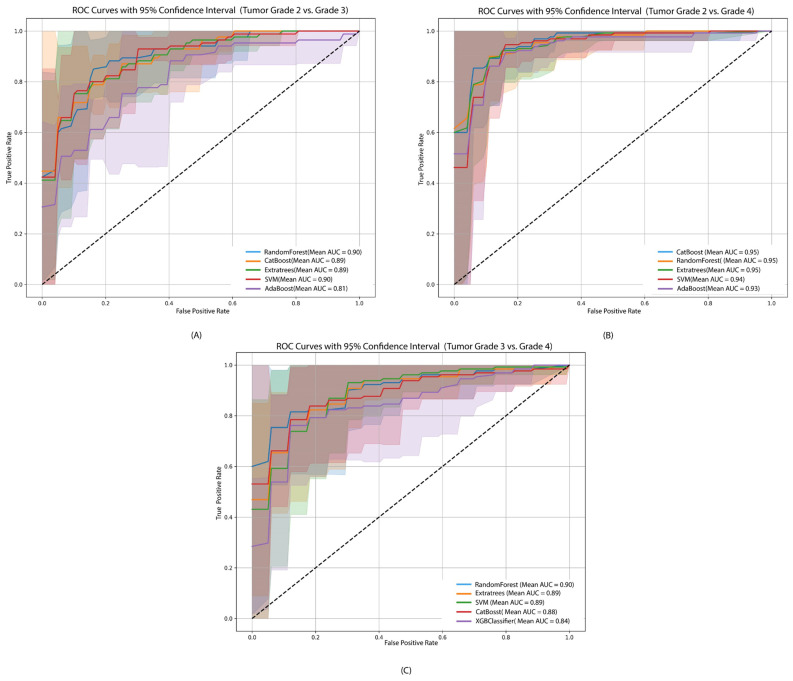
ROC curves with 95% confidence intervals for the top five performing machine learning models for the three disease classification scenarios. (**A**) Tumor grade 2 vs. grade 3, (**B**) tumor grade 2 vs. grade 4, and (**C**) tumor grade 3 vs. grade 4. The ROC curves shown correspond to the top five models identified based on their classification performance.

For tumor grade classification (Figure 5), Random Forest achieved the highest AUC for grades 2 vs. 3 (0.90) and grades 3 vs. 4 (0.90), while CatBoost and ExtraTrees led in grades 2 vs. 4 (0.95). Across all grade comparisons, ensemble-based models consistently outperformed SVM, AdaBoost, and XGBoost, confirming their robustness for tumor grade prediction.

### 3.4. SHAP Analysis

**Figure 6:**
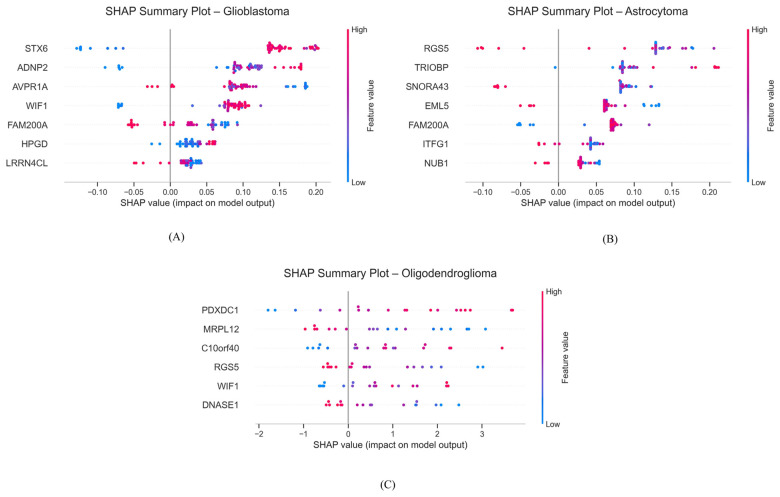
SHAP summary plots for the best-performing models across the three binary classification scenarios. (**A**) Control vs. glioblastoma, (**B**) control vs. astrocytoma, and (**C**) control vs. oligodendroglioma. The plots rank genes based on their mean absolute SHAP values, illustrating their overall impact on the prediction task. Positive SHAP values (points on the right) indicate that a feature contributes to predicting the tumor class, while negative values (points on the left) push the prediction toward the control class. The color gradient from blue to red represents the actual feature values, where blue denotes lower values, and red indicates higher values. Features with high SHAP values and red coloration on the right side of the plot contribute most strongly to the model’s classification decision.

SHAP summary plots were generated to interpret the contributions of individual genes (features) in both disease and tumor grade classification. For disease classification (Figure 6), a few subsets of genes consistently had the strongest impact on model prediction for each glioma: *STX6* and *WIF1* drove glioblastoma classification, *RGS5* and *TRIOBP* were influential for astrocytoma, and *PDXDC1* and *MRPL12* contributed most to oligodendroglioma predictions. Conversely, genes such as *FAM200A* and *LRRN4CL* for glioblastoma, *FAM200A*, *ITFG1*, and *NUB1* for astrocytoma, and *DNASE1* for oligodendroglioma had negative or minimal contributions, reflecting their relatively lower influence on model predictions.

**Figure 7:**
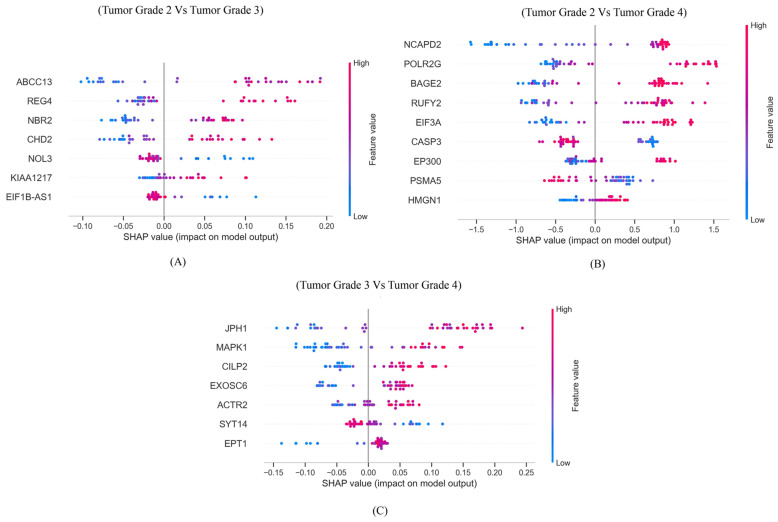
SHAP summary plots showing top predictive genes for tumor grade classification. (**A**) Tumor grade 2 vs. grade 3, (**B**) tumor grade 2 vs. grade 4, and (**C**) tumor grade 3 vs. grade 4. The plots rank genes based on their mean absolute SHAP values, illustrating their overall impact on the prediction task. Positive SHAP values (points on the right) indicate that a feature contributes to predicting the tumor class, while negative values (points on the left) push the prediction toward the control class. The color gradient from blue to red represents the actual feature values, where blue denotes lower values, and red indicates higher values. Features with high SHAP values and red coloration on the right side of the plot contribute most strongly to the model’s classification decision.

For tumor grade classification (Figure 7), SHAP analysis highlighted genes whose high expression strongly associated with higher grades (grades 3 and 4). For example, *REG4*, *NBR2*, and *CHD2* were influential for grade 3. Similarly, in panel (B), genes like *BAGE2*, *RUFY2*, and *EIF3A* are influential in classifying grade 4 tumors, while in panel (C), *ACTR2*, *SYT14*, and *EPT1* demonstrate strong positive contributions toward grade 4 prediction.

### 3.5. Box Plots

**Figure 8:**
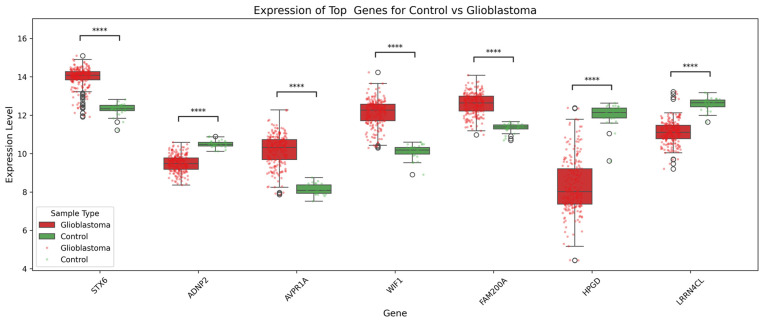
Box plots showing the differential expression of top candidate genes in glioblastoma vs. control samples. *Note:* **** = *p* ≤ 0.0001 meaning statistical difference is highly significant.

Box plots were generated to illustrate the expression levels of key genes across tumor types. In glioblastoma, *STX6*, *AVPR1A*, *WIF1*, and *FAM200A* were significantly upregulated compared to controls (*p* < 0.0001), suggesting potential oncogenic roles, while *ADNP2*, *HPGD*, and *LRRN4CL* were downregulated, suggesting potential tumor-suppressive functions. *STX6* and *WIF1* were among the most highly expressed genes in glioblastoma (Figure 8). In the astrocytoma samples, *RGS5*, *SNORA43*, *EML5*, *FAM200A*, *ITFG1*, and *NUB1* are significantly upregulated compared to controls, while *TRIOBP* is notably downregulated (see Supplementary Figure S6)**.** In the oligodendroglioma samples, *PDXDC1*, *MRPL12*, *C10orf40*, *WIF1*, and *DNASE1* are significantly upregulated compared to the control tissue, while *RGS5* was notably downregulated (see Supplementary Figure S7).

**Figure 9:**
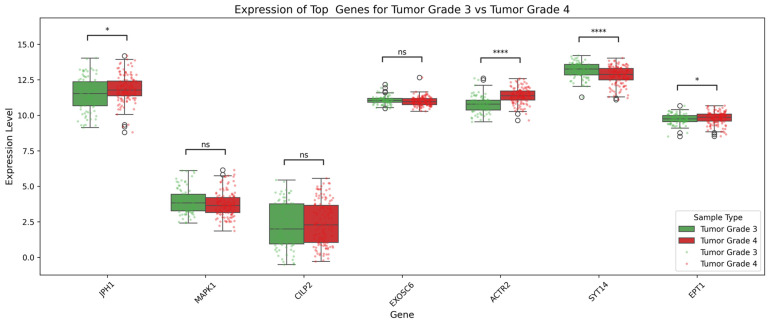
Box plots showing differential gene expression of top candidate genes between tumor grade 3 and grade 4. *Note:* **** = *p* ≤ 0.0001 meaning statistical difference is highly significant; * *p* < 0.05; ns meaning statistical difference is no significant.

Box plots illustrating differential gene expression between tumor grade 3 and grade 4 glioma samples were generated (see Figure 9). The genes analyzed include *JPH1*, *MAPK1*, *CLIP2*, *EXOSC6*, *ACTR2*, *SYT14*, and *EPT1*. Among the genes, *ACTR2* and *SYT14* show highly significant differential expression (*p* < 0.0001), suggesting a strong association with tumor grade progression. *JPH1* and *EPT1* also show statistically significant differences (*p* < 0.05), indicating potential roles in distinguishing tumor severity. On the other hand, *MAPK1*, *CLIP2*, and *EXOSC6* show no significant difference in expression, implying limited relevance for tumor grade discrimination based on their expression alone.

### 3.6. KEGG Pathway Enrichment Results

**Figure 10:**
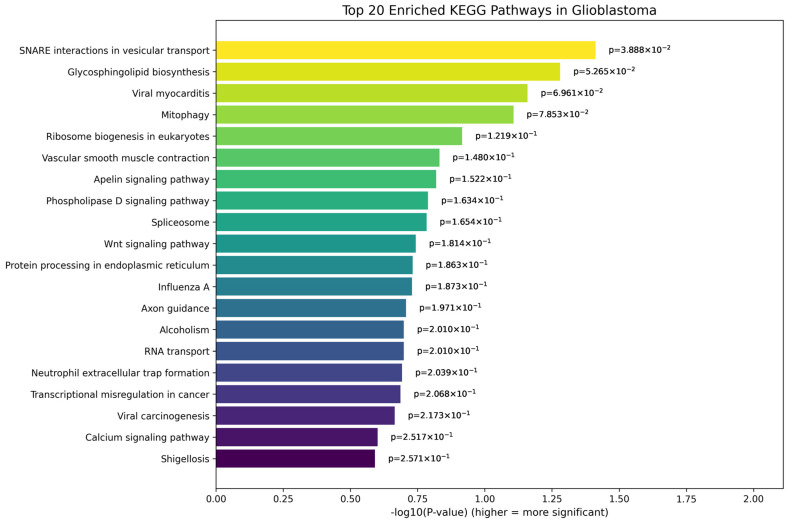
Top 20 enriched KEGG pathways for top genes in control vs. glioblastoma. The enrichment scores are visualized using −log_10_ (*p*-value), where longer bars indicate greater statistical significance (i.e., lower *p*-values).

The top 20 KEGG pathways enriched in glioblastoma compared to the control samples are shown in Figure 10. Among the top pathways, “SNARE interactions in vesicular transport” emerges as the most significantly enriched (*p* = 0.0388), followed by “Glycosphingolipid biosynthesis” and “Viral myocarditis”, possibly indicating significant alteration in vesicle-mediated transport and membrane lipid metabolism in glioblastoma tissues. These pathways are closely associated with intracellular trafficking, neurotransmitter release, and immune response mechanisms, all of which are known to be dysregulated in brain tumors. Other notably enriched pathways include “Mitophagy” and “Ribosome biogenesis”, highlighting possible disruptions in mitochondrial quality control and protein synthesis machinery; “Wnt signaling pathway” and “Spliceosome”, both of which are well-established in the regulation of tumor cell proliferation, stemness, and alternative splicing events frequently observed in glioblastoma; and “Transcriptional misregulation in cancer”, reinforcing the widespread transcriptional deregulation underlying tumor progression. The pathway analyses for the other two cases, astrocytoma and oligodendroglioma, are presented in Supplementary Figures S8 and S9, respectively.

**Figure 11:**
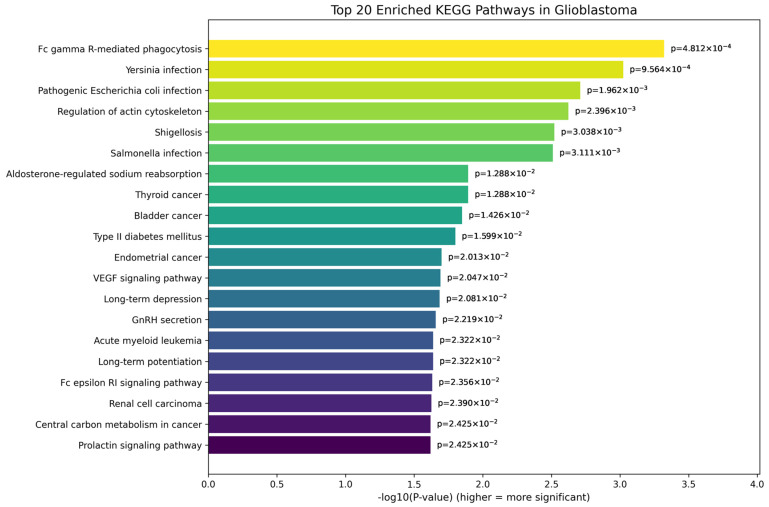
Top 20 enriched KEGG pathways for top genes in tumor grade 3 vs. tumor grade 4.

For tumor grade comparisons, the top 20 enriched KEGG pathways distinguishing grade 3 and grade 4 tumors are shown in Figure 11. Notably, immune-related pathways such as Fc gamma R-mediated phagocytosis and bacterial infection pathways (e.g., Yersinia and *Escherichia coli* infection) suggest heightened immune modulation in grade 4 tumors, particularly glioblastoma. The enrichment of pathways like regulation of actin cytoskeleton, VEGF signaling, and central carbon metabolism reflects increased cellular motility, angiogenesis, and metabolic reprogramming—hallmarks of the aggressive phenotype of grade 4 tumors. Additionally, the involvement of neurological and hormone-related pathways, such as GnRH secretion and prolactin signaling, indicates possible disruption of neuroendocrine signaling in high-grade gliomas. Collectively, these pathways underscore the complex molecular and genetic landscape distinguishing the more infiltrative and treatment-resistant grade 4 gliomas from grade 3 tumors.

## 4. Discussion

In this study, we applied multiple ML models to transcriptomic data from the REMBRANDT dataset to classify glioma subtypes and tumor grades. Using feature selection approaches and interpretable ML techniques, we identified genetic associations capable of distinguishing tumor types from normal brain tissue as well as differentiating between tumor grades. Model interpretability was further explored using SHAP analysis, allowing the identification of key genes contributing to classification decisions. Finally, gene set enrichment analysis was conducted to investigate the biological pathways associated with these predictive genes. Together, these approaches demonstrate that the proposed interpretable ML framework can accurately classify glioma subtypes and tumor grades from transcriptomic data, while SHAP analysis identifies the gene features most influential for model predictions.

Across the disease classification tasks, several ML models demonstrated strong predictive performance, with Random Forest, XGBoost, and SVM achieving near-perfect AUC values in distinguishing tumor samples from controls. These results highlight the ability of ensemble-based models to capture complex nonlinear relationships within high-dimensional transcriptomic datasets. Tree-based methods such as Random Forest and ExtraTrees are particularly well suited to gene expression analysis due to their capacity to model feature interactions and handle large numbers of predictors [26]. Importantly, the integration of SHAP analysis enabled transparent interpretation of model predictions by quantifying the contribution of individual genes to classification outcomes, addressing the common limitation of black-box ML approaches in biomedical research.

SHAP-guided feature interpretation identified several genes strongly associated with disease classification across glioma subtypes. In glioblastoma, genes such as *STX6*, *WIF1*, *AVPR1A*, and *FAM200A* were upregulated compared to the control group, suggesting potential oncogenic roles in cellular signaling, vesicular transport, and metabolic regulation. Indeed, *FAM200A* is associated with poor prognosis in GBM, though its specific biological role in glioma remains largely unexplored [34]. Syntaxin-6 promotes glioma aggressiveness by facilitating EGFRvIII nuclear transport, enhancing oncogenic signaling pathways like STAT3 and PKM2 [35]. *WIF1* acts as a tumor suppressor in GBM by inhibiting Wnt signaling, with its downregulation associated with increased tumor growth and poorer patient survival [36,37,38,39,40].

In contrast, genes including *HPGD*, *ADNP2*, and *LRRN4CL* were downregulated, which is consistent with potential tumor-suppressive functions that may become diminished during tumorigenesis. *HPGD*, for example, acts as a tumor suppressor in GBM by inhibiting prostaglandin-driven tumor growth, with higher expression linked to better patient survival [41,42]. Additionally, *ADNP2* is linked to glioma progression and malignant transformation [43], but its mechanistic role in tumor biology is still not well established. Therefore, the observed expression patterns support the biological plausibility of the ML-derived feature importance.

Distinct gene signatures were also observed for other glioma subtypes. Astrocytoma samples were characterized by an increased expression of *RGS5*, *SNORA43*, *EML5*, *ITFG1*, and *NUB1*. While the biological relevance of many of these genes in astrocytoma specifically remains incompletely defined, some of these genes have indeed been investigated. For example, *RGS5* was shown to promote inflammation and abnormal tumor blood vessel formation in astrocytomas by enhancing TNF signaling in astrocytes and being upregulated by hypoxia in tumor vasculature [44,45]. Conversely, *TRIOBP* was downregulated relative to the control group. In oligodendroglioma, genes such as *PDXDC1*, *MRPL12*, *C10orf40, WIF1*, and *DNASE1* were significantly upregulated, suggesting involvement of mitochondrial metabolism, transcriptional regulation, and cellular homeostasis in subtype-specific tumor biology. Although these genes have not been specifically studied in oligodendroglioma, their established roles in cancer-related processes (e.g., *MRPL12* and *C10orf40* in tumor progression [46,47,48,49]) suggest potential relevance and warrant further investigation.

Beyond subtype classification, our analyses also revealed gene expression patterns associated with glioma progression across tumor grades. In particular, genes such as *ACTR2*, *SYT14*, and *EPT1* demonstrated strong positive contributions to the prediction of higher-grade tumors. *ACTR2* plays a role in actin cytoskeleton remodeling, a key process underlying cellular migration and invasion [50,51], while *SYT14* is involved in vesicular trafficking and neurotransmitter release mechanisms [52,53,54]. *SYT14* is notably overexpressed in glioma cells, and its knockdown has been shown to reduce cell proliferation and colony formation, increase apoptosis, and disrupt normal cell cycle progression, suggesting a potential pathogenic role in glioma pathogenesis [55]. The upregulation of these genes in higher tumor grades may therefore reflect increased cellular motility and aggressive tumor behavior. Similarly, *JPH1* and *EPT1* showed statistically significant differences between grade 3 and grade 4 tumors, suggesting a potential involvement in glioma progression.

Other genes analyzed in the grade classification task, including *MAPK1*, *CLIP2*, and *EXOSC6*, displayed relatively stable expression across grades and showed limited discriminative power. This observation underscores that not all transcriptional alterations contribute equally to tumor progression and highlights the importance of feature selection and interpretability methods in identifying biologically meaningful markers. Overall, the genes associated with tumor grade differentiation converge on biological processes linked to cellular motility, intracellular transport, and metabolic regulation, which are known hallmarks of high-grade glioma aggressiveness.

Pathway enrichment analysis further supported these findings by revealing biological processes associated with the identified genes. In glioblastoma, enriched pathways included SNARE interactions in vesicular transport, glycosphingolipid biosynthesis, and mitophagy, raising the possibility of alterations in intracellular trafficking, membrane lipid metabolism, and mitochondrial quality control. Additional pathways, such as ribosome biogenesis, the spliceosome, and transcriptional misregulation in cancer, highlight widespread disruptions in gene expression regulation and protein synthesis machinery in glioblastoma cells. The enrichment of the Wnt signaling pathway further supports the possible involvement of key oncogenic signaling mechanisms in glioma development.

For tumor grade progression, enriched pathways included Fc gamma receptor-mediated phagocytosis, regulation of actin cytoskeleton, VEGF signaling, and central carbon metabolism, reflecting increased immune modulation, cellular motility, angiogenesis, and metabolic reprogramming in high-grade tumors. The involvement of neuroendocrine signaling pathways, such as GnRH secretion and prolactin signaling, raises the hypothesis of additional regulatory disruptions that may contribute to glioma progression. Collectively, these pathway-level findings provide functional context for the gene signatures identified through SHAP analysis and further emphasize the complex molecular mechanisms underlying glioma aggressiveness.

Despite these promising findings, several limitations should be acknowledged. First, the moderate sample size and inherent variability of retrospective transcriptomic datasets may limit the generalizability of the findings, highlighting the need for larger multi-cohort analyses. Second, this study utilized the REMBRANDT dataset, which relies on earlier histopathological classifications. Since the publication of the WHO fifth edition classification of central nervous system tumors (WHO CNS5, 2021), glioma taxonomy has shifted toward integrated molecular diagnostics, incorporating key biomarkers such as *IDH* mutation and 1p/19q codeletion [56]. Because the REMBRANDT dataset predates WHO CNS5, it lacks essential molecular markers, most notably *IDH1/IDH2* mutation status and 1p/19q codeletion, that now define the major diagnostic categories of adult-type diffuse gliomas. As a result, modern entities such as astrocytoma (*IDH*-mutant) and oligodendroglioma (*IDH*-mutant, 1p/19q-codeleted) cannot be reliably reconstructed from the available labels, and some samples classified histologically as glioblastoma, astrocytoma, or oligodendroglioma may not align with their molecularly defined contemporary equivalents.

Third, class imbalance within the REMBRANDT dataset represents an important methodological limitation. Although SMOTE-Tomek was applied to mitigate the imbalance by generating synthetic minority samples and removing borderline Tomek links, this approach may influence biological interpretability. Synthetic oversampling can introduce gene-expression patterns that do not perfectly reflect true biological variability, potentially altering feature importance rankings or exaggerating the contribution of specific genes. While SMOTE-Tomek improves classifier performance in imbalanced settings, the resulting models should therefore be interpreted cautiously, as the synthetic samples may not capture the full molecular heterogeneity of real glioma subtypes and grades. Future work incorporating larger, balanced, and molecularly annotated cohorts will be essential to confirm that the predictive features identified truly represent biologically meaningful signals rather than artifacts of oversampling.

Finally, although the models demonstrated strong predictive performance using cross-validation, external validation using independent cohorts such as The Cancer Genome Atlas (TCGA) was not performed [57,58]. Additionally, prospective evaluation using unlabeled clinical samples analyzed in a blinded manner and comparing model predictions to gold standard pathological diagnosis will be necessary to assess real-world performance. Future work incorporating modern WHO-aligned datasets, such as TCGA-GBM and TCGA-LGG, will also be essential to validate the clinical applicability of the predictive models under the WHO CNS5 classification framework.

## 5. Conclusions

In conclusion, in the present study, we applied multiple machine learning models to transcriptomic data from the REMBRANDT dataset to classify glioma subtypes and tumor grades. The models achieved high predictive performance, with Random Forest and XGBoost showing particular utility for these classification tasks. SHAP analysis identified genes that contributed most to model predictions (e.g., *WIF1*, *STX6*, *RGS5*, and *ACTR2*) and pathway enrichment analysis revealed candidate biological processes associated with the selected features. These findings are statistical in nature and will require further biological experimental validation. Independent validation on more recent, molecularly annotated datasets will also be necessary to assess the generalizability and clinical applicability of the proposed ML framework.

## Figures and Tables

**Table 1:** The performance metrics of machine learning models for disease classification (gliomas vs. controls). The models were evaluated using key metrics including accuracy, precision, recall, specificity, F1-score, and AUC.

**Control vs. Glioblastoma**						
Models	Accuracy (%)	Precision (%)	Recall (%)	Specificity (%)	F1-score (%)	AUC (%)
Random Forest	99.59839	99.61224	99.59839	99.59839	99.60146	99.89
XGBClassifier	98.39357	98.59438	98.39357	98.39357	98.43979	99.92
Logistic Regression	97.99197	98.29622	97.99197	97.99197	98.06278	99.89
KNeighbors	96.78715	97.50112	96.78715	96.78715	96.95834	98.42
AdaBoost	95.18072	95.55272	95.18072	95.18072	95.31936	95.66
**Control vs. Astrocytoma**						
Models	Accuracy (%)	Precision (%)	Recall (%)	Specificity (%)	F1-score (%)	AUC (%)
Random Forest	98.29545	98.33077	98.29545	98.29545	98.30752	99.35
XGBClassifier	97.72727	98.01136	97.72727	97.72727	97.78746	99.83
LinearDiscriminantAnalysis	97.15909	97.58953	97.15909	97.15909	97.25112	98.62
MLPClassifier	96.59091	96.72686	96.59091	96.59091	96.63809	98.77
AdaBoost	95.45455	95.62059	95.45455	95.45455	95.51745	94.32
**Control vs. Oligodendroglioma**						
Models	Accuracy (%)	Precision (%)	Recall (%)	Specificity (%)	F1-score (%)	AUC (%)
SVM	98.94737	98.98367	98.94737	99.56009	98.95264	99.89
Random Forest	97.89474	98.03509	97.89474	99.12019	97.91509	99.84
Logistic Regression	96.84211	97.14771	96.84211	98.68028	96.88623	100
LinearDiscriminantAnalysis	93.68421	94.79876	93.68421	97.36057	93.84179	100
LGBM	90.52632	90.64401	90.52632	87.72528	90.5738	98.29

**Table 2:** The performance metrics of the machine learning models for tumor grades classification (grades 2, 3, and 4). The models were evaluated using key metrics including accuracy, precision, recall, specificity, F1-score, and AUC.

**Tumor Grade 2 vs. Tumor Grade 3**						
Models	Accuracy (%)	Precision (%)	Recall (%)	Specificity (%)	F1-score (%)	AUC (%)
Random Forest	83.69565	83.70555	83.69565	83.17303	83.66064	88.21
CatBoost	80.97826	81.04912	80.97826	80.17444	80.89458	87.71
ExtraTrees	80.43478	80.4678	80.43478	79.70782	80.36461	87.99
SVM	80.43478	80.41663	80.43478	80.04056	80.41615	87.72
AdaBoost	71.73913	71.70231	71.73913	71.24364	71.71222	79.36
**Tumor Grade 2 vs. Tumor Grade 4**						
Models	Accuracy (%)	Precision (%)	Recall (%)	Specificity (%)	F1-score (%)	AUC (%)
CatBoost	91.26638	91.25812	91.26638	90.69944	91.25515	94.76
Random Forest	90.82969	90.82508	90.82969	90.12602	90.81146	94.83
ExtraTrees	90.39301	90.39301	90.39301	90.03434	90.39301	94.68
SVM	88.64629	88.64629	88.64629	88.2224	88.64629	92.87
AdaBoost	87.77293	87.76085	87.77293	86.83469	87.7395	91.79
**Tumor Grade 3 vs. Tumor Grade 4**						
Models	Accuracy (%)	Precision (%)	Recall (%)	Specificity (%)	F1-score (%)	AUC (%)
Random Forest	84.18605	84.48431	84.18605	83.95875	84.26882	90.81
ExtraTrees	82.32558	82.40861	82.32558	81.11333	82.35983	88.99
SVM	81.86047	81.99568	81.86047	80.80922	81.91187	88.63
CatBoost	80.93023	80.9717	80.93023	79.38651	80.94918	88.52
XGBClassifier	79.06977	79.48696	79.06977	78.57729	79.19409	83.53

## Data Availability

The gene expression dataset analyzed in this study is publicly available in the Gene Expression Omnibus (GEO) under accession number GSE108476 (REMBRANDT dataset).

## Supplementary Materials

The following supporting information can be downloaded at https://www.mdpi.com/article/10.3390/genes17050511/s1. Figure S1: t-SNE Visualization of disease groups and Control Samples Using Top-Ranked Gene Expression Features, Figure S2: t-SNE Visualization of tumor grade Samples Using Top-Ranked Gene Expression Features, Figure S3: Hierarchical cluster heatmap for control vs. glioblastoma using top genes, Figure S4: Hierarchical cluster heatmap for control vs. astrocytoma using top genes, Figure S5: Hierarchical cluster heatmap for control vs. oligodendroglioma using top genes, Figure S6: Differential expression of top candidate genes in control vs. astrocytoma samples, Figure S7: Differential expression of top candidate genes in control vs. oligodendroglioma samples, Figure S8: Top 20 enriched KEGG pathways for top genes in control vs. astrocytoma, Figure S9: Top 20 enriched KEGG pathways for top genes in Control vs. Oligodendroglioma.
