# Supplementary material for: Machine Learning-Based Classification of Gliomas and Tumor Grades with SHAP-Guided Feature Interpretation
Source: Genes (Basel). 2026 Apr 25;17(5):511. doi: 10.3390/genes17050511 (PMC13205932; doi:10.3390/genes17050511)
Supplement: Supplementary file 1 [file genes-17-00511-s001.zip › genes-4263181-supplementary.pdf]

Supplementary Materials

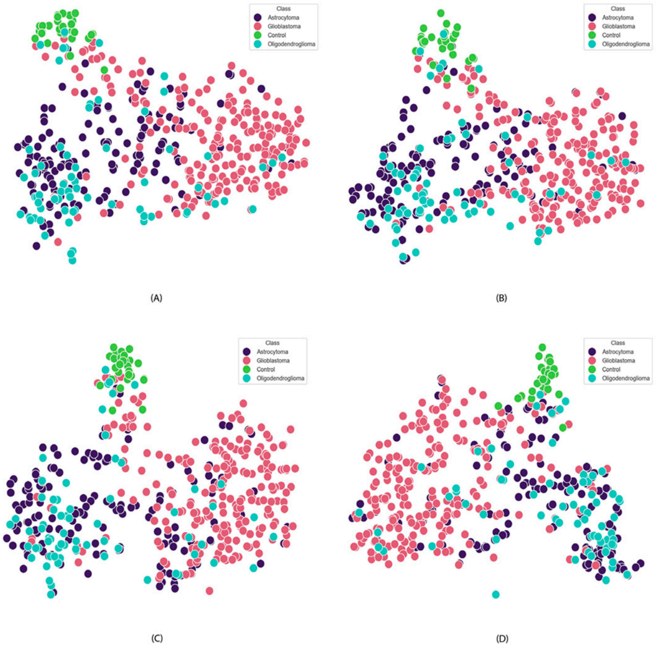

Figure S1*. t-SNE Visualization of disease groups and Control Samples Using Top-Ranked Gene Expression Features.* (A) Top 40 features, (B) Top 30 features, (C) Top 20 features, (D) Top 10 features. Each dot represents a sample, color-coded by class: astrocytoma (dark blue), glioblastoma (pink), oligodendroglioma (light green), and control (cyan). As the number of selected features decreases, the clustering structure becomes less defined, though core groupings remain visible. Notably, glioblastoma and astrocytoma exhibit overlapping regions, consistent with their shared lineage, while oligodendroglioma and control samples tend to form more discrete clusters. These t-SNE plots underscore the effect of dimensionality reduction and feature selection in revealing the underlying transcriptomic distinctions among brain tumor subtypes.

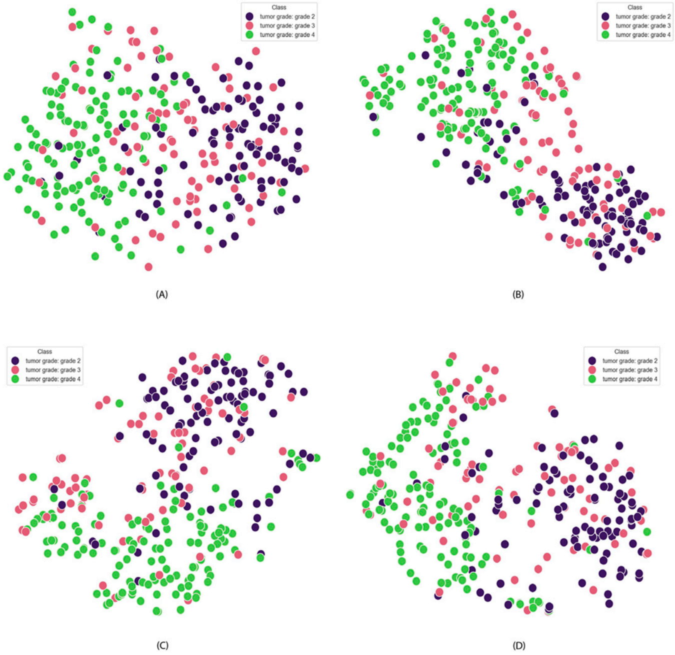

Figure S2. *t-SNE Visualization of tumor grade Samples Using Top-Ranked Gene Expression Features.* (A) Top 40 features, (B) Top 30 features, (C) Top 20 features, (D) Top 10 features. Subfigures (A) through (D) correspond to models built on the top 40, top 30, top 20, and top 10 most informative features, respectively. As evident in the plots, samples tend to cluster according to their tumor grade, with greater separation and clearer boundaries when a higher number of features is used (A and B). As the feature count is reduced (C and D), the boundaries between grades become less distinct, particularly between grades 2 and 3, suggesting overlap in molecular profiles at intermediate stages. Notably, grade 4 tumors consistently form a more distinguishable cluster, reflecting their more aggressive and distinct transcriptomic signature.

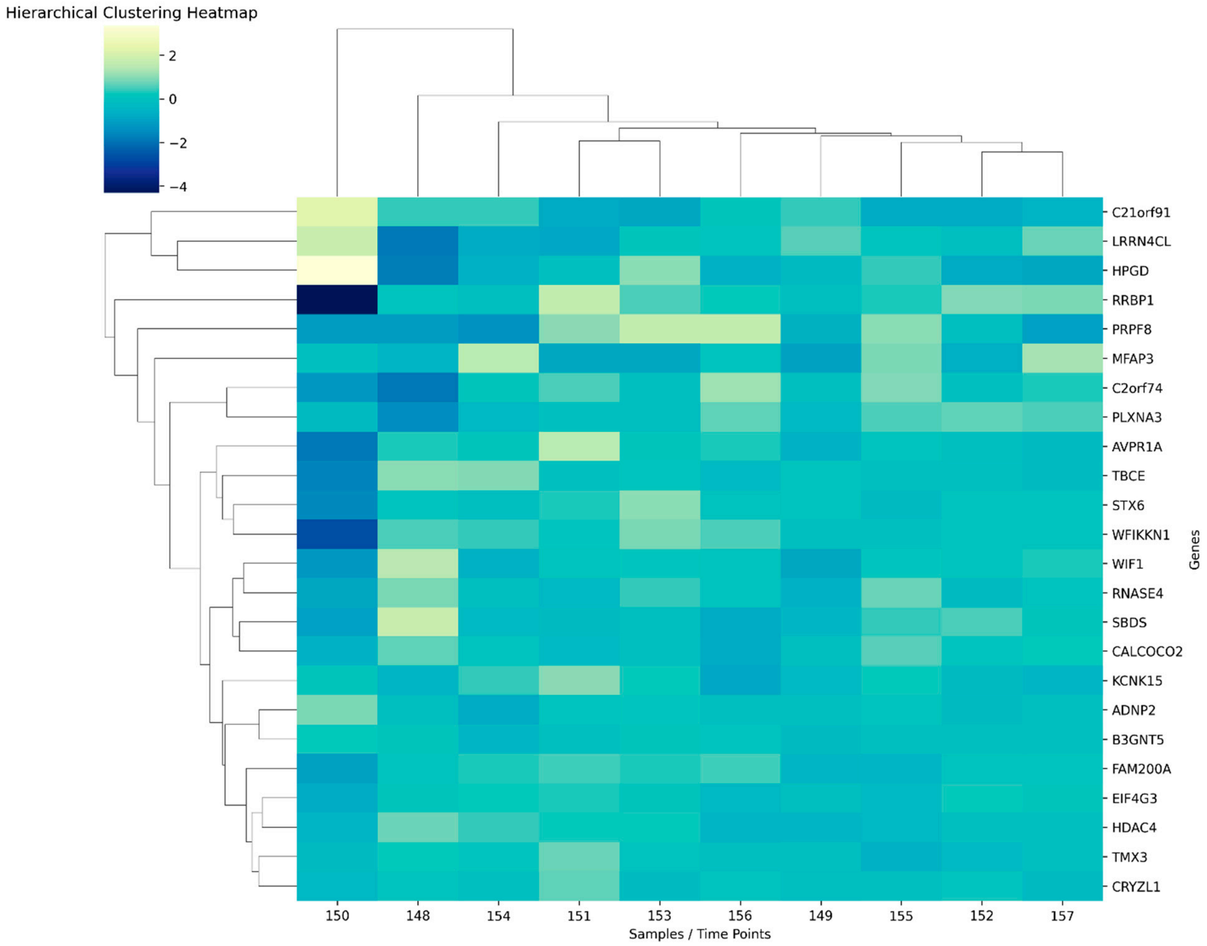

Figure S3. *Hierarchical Cluster Heatmap for Control vs. Glioblastoma Using Top Genes.*

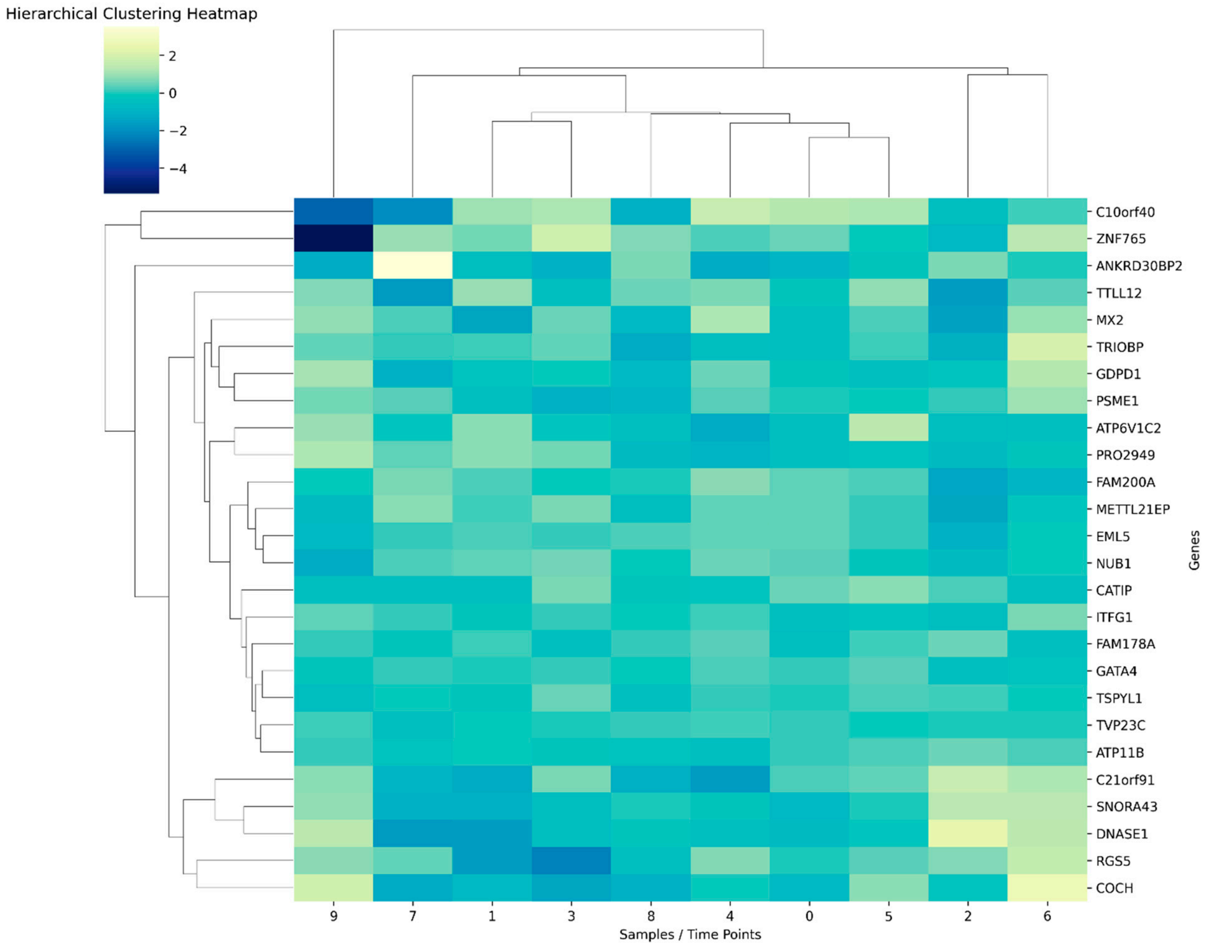

Figure S4. *Hierarchical Cluster Heatmap for Control vs. Astrocytoma Using Top Genes.*

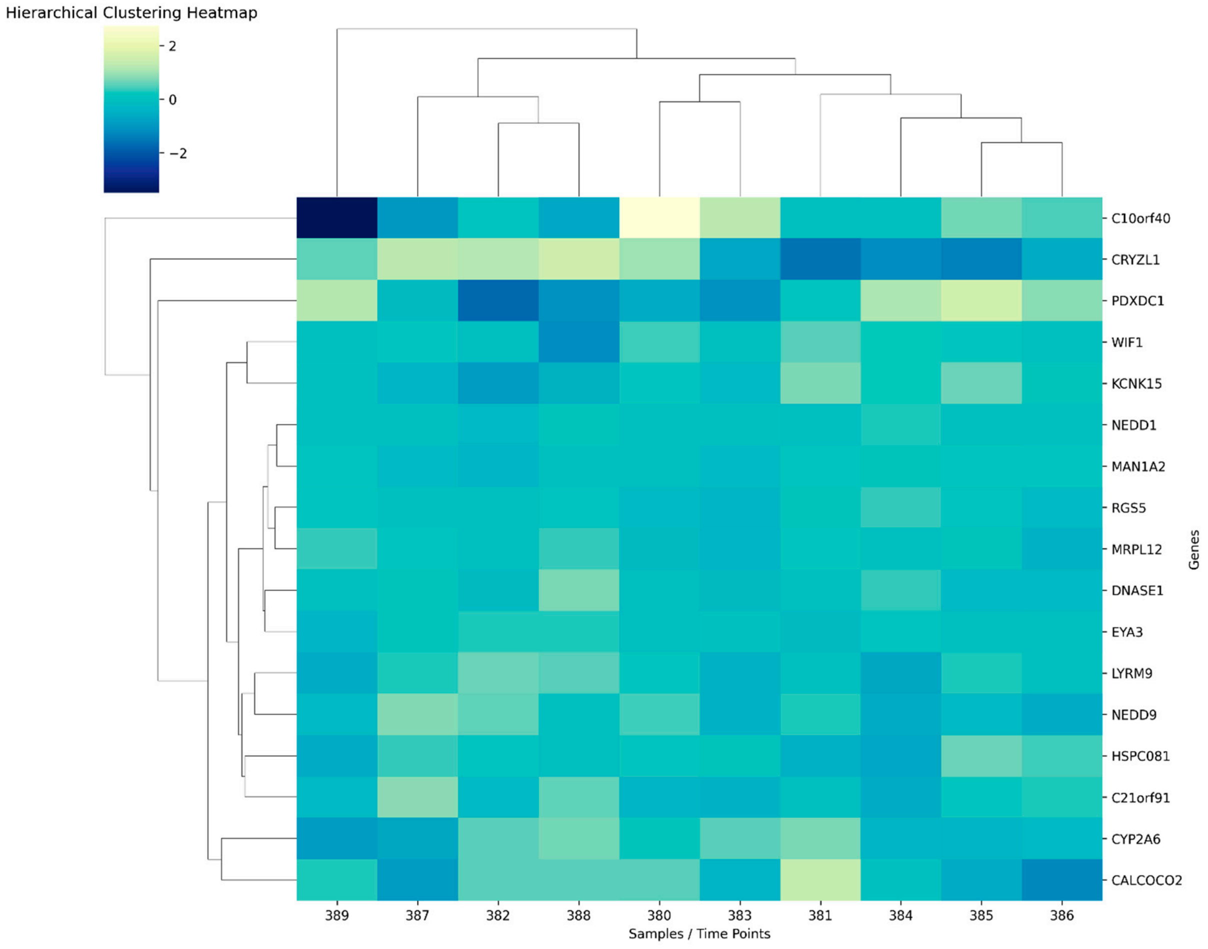

Figure S5. *Hierarchical Cluster Heatmap for Control vs. Oligodendroglioma Using Top Genes.*

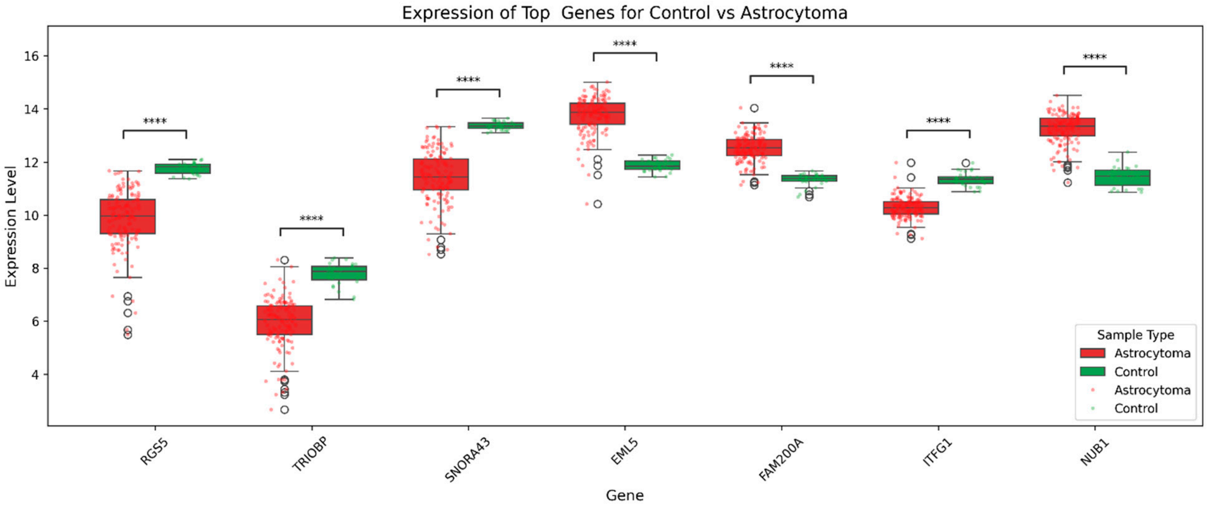

Figure S6. *Differential Expression of Top Candidate Genes in Control vs. Astrocytoma Samples.*

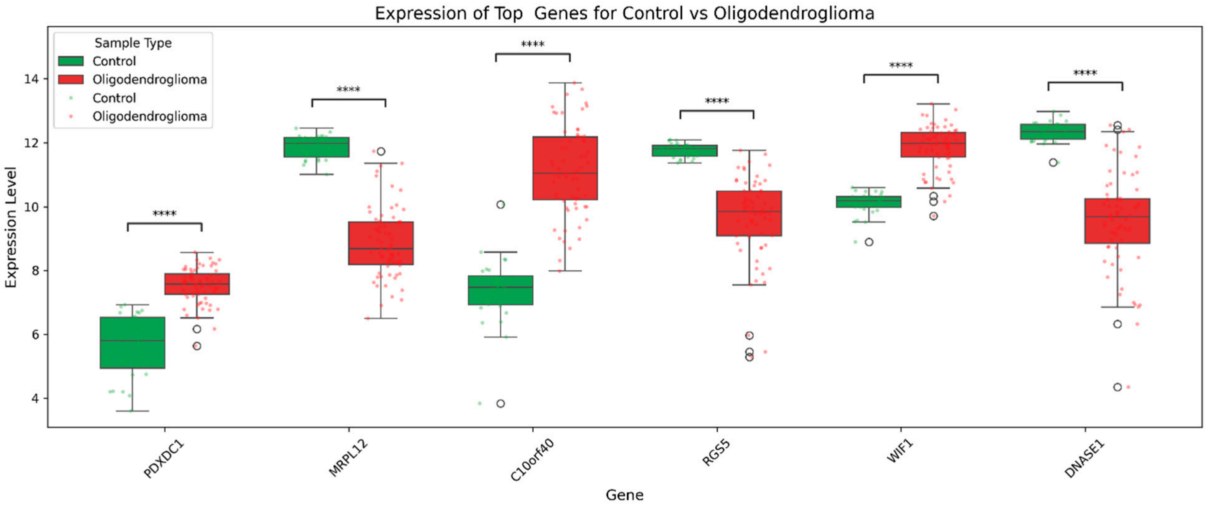

Figure S7. *Differential Expression of Top Candidate Genes in Control vs. Oligodendroglioma Samples.*

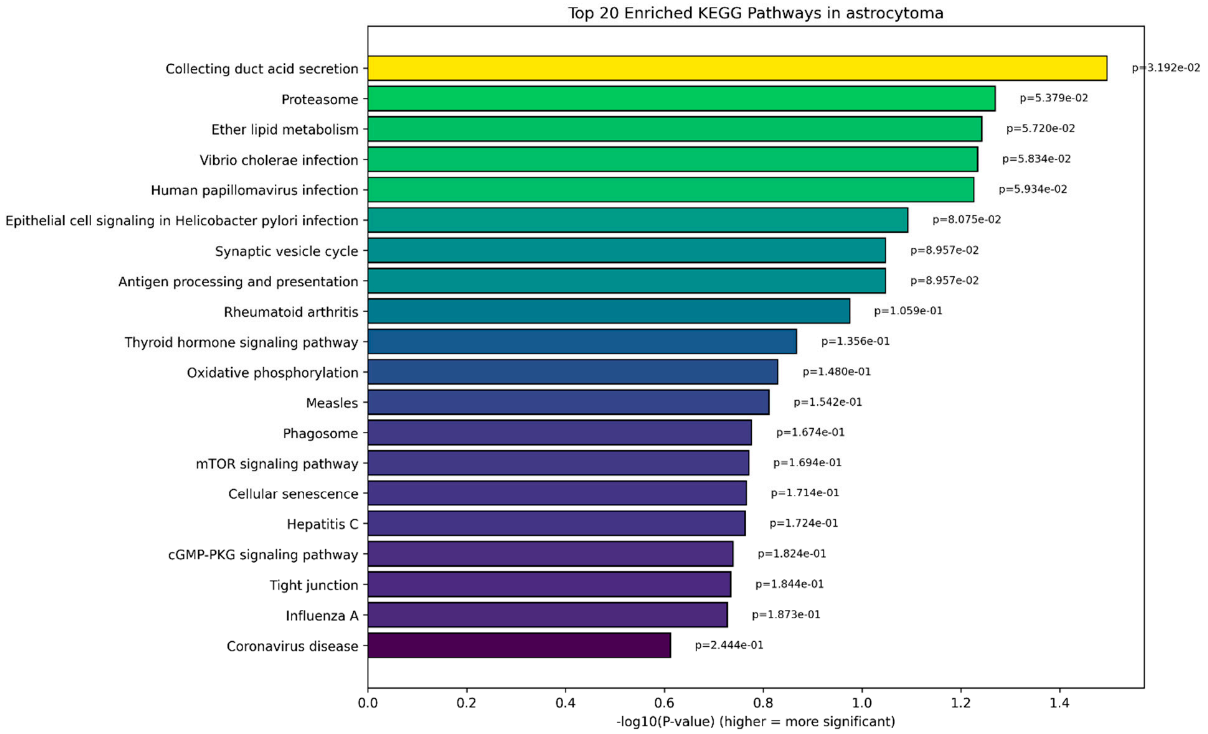

Figure S8. *Top 20 enriched KEGG pathways for top genes in Control vs. Astrocytoma.*

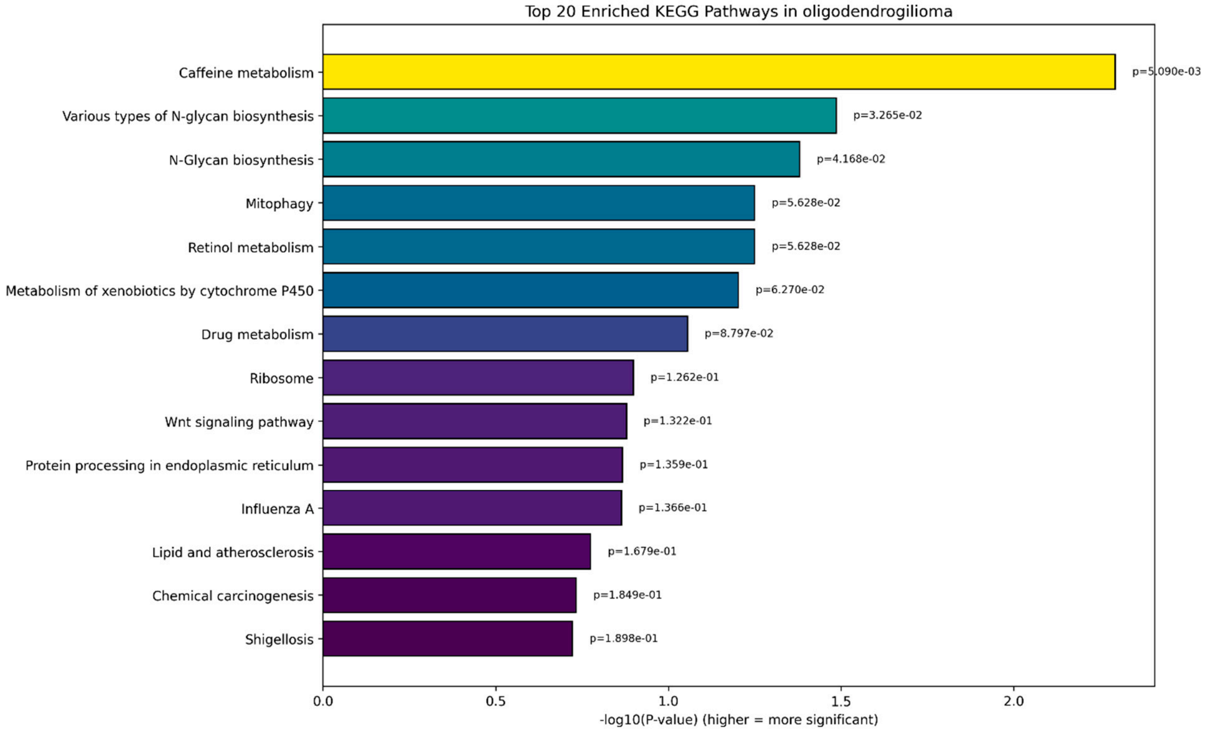

Figure S9. *Top 20 enriched KEGG pathways for top genes in Control vs. Oligodendroglioma.*

Evaluation Metrics

Equations S1 to S5 present the mathematical definitions of the five evaluation metrics: weighted recall (sensitivity), specificity, precision, overall accuracy, and F1 score.

$$\text{Accuracy}_{\text{class}_j} = \frac{TP_j+TN_j}{TP_j+TN_j+FP_j+FN_j} \tag{S1}$$

$$\text{Precision}_{\text{class}_j} = \frac{TP_j}{TP_j+FP_j} \tag{S2}$$

$$\text{Recall}_{\text{class}_j} = \frac{TP_j}{TP_j+FN_j} \tag{S3}$$

$$\text{F1\_score}_{\text{class}_j} = 2 \times \frac{\text{Precision}_j \times \text{Recall}_j}{\text{Precision}_j + \text{Recall}_j} \tag{S4}$$

$$\text{Specificity}_{\text{class}_j} = \frac{TN_j}{TN_j+FP_j} \tag{S5}$$

The mathematical formulations of the five evaluation metrics—weighted recall (sensitivity), specificity, precision, overall accuracy, and F1 score—are provided in Equations S1 through S5.
